# Spatial diversity and distribution of fern and lycophyte species in karst and non-karst landscapes towards conservation needs

**DOI:** 10.3389/fpls.2025.1495796

**Published:** 2025-03-03

**Authors:** Marjorie D. delos Angeles, Sirilak Radbouchoom, Boniface K. Ngarega, R. Sedricke Lapuz, Harald Schneider

**Affiliations:** ^1^ Center for Integrative Conservation and Yunnan Key Laboratory for Conservation of Tropical Rainforests and Asian Elephants, Xishuangbanna Tropical Botanical Garden, Chinese Academy of Sciences, Mengla, Menglun, Yunnan, China; ^2^ University of Chinese Academy of Sciences, Beijing, China; ^3^ Plant Biology Division, Institute of Biological Sciences, College of Arts and Sciences, University of the Philippines Los Baños, Los Baños, Philippines; ^4^ Department of Botany, Jomo Kenyatta University of Agriculture and Technology, Nairobi, Kenya; ^5^ School of Biological Sciences, The University of Hong Kong, Hong Kong, Hong Kong SAR, China

**Keywords:** diversity hotspots, gap analysis, multisource data, Philippines, pteridophytes

## Abstract

Karst formations are distinguished by their high levels of species diversity and endemism, including ferns and lycophytes. However, the existing data on plant community composition in karst formations remains deficient. Addressing these knowledge gaps is imperative, given the current accelerated rates of species loss, to enhance efforts to conserve biodiversity in these habitats. This study documents and explains patterns of fern and lycophyte species diversity within karst landscapes (KL) and non-karst landscapes (NKL) in the Philippines. Our comprehensive analysis involved aggregating 19,529 occurrence points encompassing 1,024 fern and lycophyte species sourced from field expeditions, voucher records from local herbaria, and online databases. Indices for species richness, weighted endemism, and corrected weighted endemism were then computed across KL and NKL areas to describe spatial diversity and identify fern and lycophyte hotspot areas. Gap analyses were also performed to determine if established protected areas (PAs) were sufficient to cover the identified fern and lycophyte diversity hotspots. Principal Component Analysis (PCA) was conducted to determine potential ecological drivers of distribution between KL and NKL areas. The findings reveal that most fern and lycophyte species were recorded to occur in NKL areas, with 995 (97.16%) species identified, while 676 (66.02%) species were documented to occur in KLs, including 29 (2.83%) exclusive to karsts. Identified hotspots for NKL are within mountain ecosystems, which are already under existing legal protection. In contrast, KLs have five areas identified as congruent hotspots but considered gap areas due to their exclusion from current PA boundaries. Existing PAs thus provide less protection to karst habitats and their associated floras. PCA did not reveal any significant environmental predictors, suggesting separation of KL and NKL species distributions, possibly due to lack of high-resolution environment data available for karsts. To facilitate the conservation of fern and lycophyte species in karsts, we propose installing and expanding existing PA boundaries, along with conducting more focused surveys in karst regions to better understand their ecological dynamics.

## 1 Introduction

The continuous overexploitation of the world’s natural resources takes global biodiversity towards the sixth mass extinction (Ceballos et al., 2017; Cowie et al., 2022), highlighting the need for prioritizing the conservation of threatened species and ecologically important ecosystems (Clements et al., 2006). Effective conservation of species and habitats, however, cannot be achieved without the necessary data, and it is important to delineate and identify hotspot areas to guide conservation efforts (Myers et al., 2000).

Karst regions, similar to islands, represent an important source of regional biodiversity and endemism (Geekiyanage et al., 2019), providing a wide range of microhabitats due to its highly structured geomorphology resulting from the interaction of water and wind with limestone carbonates (Clements et al., 2006; Tolentino et al., 2020). Karst landscapes are characterized by their fragility, edaphic complexity, and harsh natural ecological environments due to their thin soils that are deficient in nitrogen and phosphorus and have high concentrations of calcium and magnesium (Yang et al., 2022; The Long and Dac Trien, 2019; Geekiyanage et al., 2019). Given these characteristics, karst landscapes provide unique challenges to the plants colonizing them (Green et al., 2019; Liu et al., 2021). Despite hosting several highly specialized species with restricted distribution ranges, karst areas are often overlooked habitats in biodiversity protection efforts. The karst regions of Southeast Asia have particularly been flagged out as a challenge to biodiversity conservation (Clements et al., 2006). While these habitats are rich in species highly dependent on the unique characters of karst landscapes (Ford and Williams, 2007; Fu et al., 2022), they also experience threats from mining activities and the impacts of global climate change (Whitten, 2012; Wong et al., 2016; He et al., 2021). These threats have contributed to increasing species extinctions (Phutthai and Hughes, 2017), highlighting the critical need for comprehensive biological data collection in karst landscapes (Salas et al., 2005, Bystriakova et al., 2019; Fu et al., 2022; Molina-Paniagua et al, 2023).

Surveying plants occurring on tropical karst regions is therefore considered as a priority. This is crucial to support the establishment of protected areas, which target the conservation of species endemic to tropical karst formations. The enhanced availability of digitized historical collections, such as herbarium specimens stored in databases such as the Global Biodiversity Information Facility (GBIF), has recently allowed the assessment of species distribution across different landscapes (Bystriakova et al., 2019; Ramírez-Barahona et al., 2023). Given the recent access to big spatial data, metrics such as species richness, weighted endemism, corrected weighted endemism, and beta diversity have been utilized to determine biodiversity hotspots (Yu et al., 2017; Chen et al., 2022). These have also been used to perform gap analyses in existing conservation measures and protected areas for their possible enhancement and area expansions (Myers, 1988; Scott et al., 1993; Jennings, 2000; Myers et al., 2000; Xu et al., 2017; Brummitt et al., 2021).

Despite challenges brought about by karst landscapes, it can support ferns and lycophytes. These ancient linages with approximately 12,000 recognized species globally (PPG1, 2016) account for 3% of the world’s vascular plant diversity and rank second in diversity after angiosperms (Kress, 1986; Schuettpelz and Pryer, 2009; Nitta et al., 2022). They play pivotal roles in tropical terrestrial ecosystems (Salovaara et al., 2004; Mehltreter, 2010; Watkins and Cardelús, 2012; Bergeron and Pellerin, 2014; Pouteau et al., 2016; Silva et al., 2018), where they exist as terrestrial, epiphytic, and as lithophytic organisms (Page, 2002; Mehltreter, 2010; Watkins et al., 2010). Calcareous ferns were recorded to exhibit high diversity within the families Pteridaceae, Aspleniaceae, and Dyopteridaceae in select Mexican limestone regions (Flores-Galván et al., 2024).

Ferns and lycophytes are sensitive to changes in the environment, making them valuable bioindicators of terrestrial ecosystem health. While their potential as bioindicators have been well explored and have been used in classifying forest types (Salovaara et al., 2004) and determining forest integrity (Bergeron and Pellerin, 2014), their application as bioindicators in tropical karst landscapes remains underutilized. Furthermore, Karst microhabitats, characterized by unique geological formations and microclimates, can act as refugia for site-endemic species. This assertion is supported by the successful recovery of numerous locally endemic fern species within these environments (Takeuchi, 2007; Brownsey and de Lange, 1997; He and Zhang, 2010; Yesilyurt and Schneider, 2010; Tang et al., 2017). The investigation of fern and lycophyte assemblages in limestone forests, particularly on habitats formed over karst formations, remains a relatively understudied aspect of botanical research in the tropics, including the Philippines. Existing studies on ferns and lycophytes of karst landscapes in the country are predominantly centered on enumerations and are primarily taxonomic driven (Price, 1975; Iwatsuki and Price, 1977; Zamora and Co, 1986; Barcelona, 2003; Barcelona et al., 2006; delos Angeles et al., 2022; delos Angeles and Asis, 2022). There is also limited information on drivers of their distribution across both landscapes.

To bridge this knowledge gap, this study therefore undertook a comprehensive analysis of fern and lycophyte diversity, prevalent in both karst and non-karst landscapes within the Philippines and explore the influence of edaphic and bioclimatic factors on their assemblages. To achieve these, we compiled a dataset to illustrate the spatial distributions of ferns and lycophytes across both landscapes. We also performed gap analyses focused on linking the identified biodiversity hotspots in karst and non-karst habitats with existing protected areas. Finally, we propose a list of hotspots on karst areas to be considered as candidates for newly established national parks.

## 2 Materials and methods

### 2.1 Study area

The Philippines, an archipelago of more than 7,600 islands, harbors multiple centers of endemism (Mittermeier et al., 2004). A significant proportion of the country’s land area is characterized by karst formations, which are developed over limestone deposits composed by tertiary and quaternary carbonates (Balázs, 1973). Specifically, approximately 10% of the Philippines I covered by limestone forest formations (Piccini and Rossi, 1994; Balázs, 1973).

#### 2.1.1 Assembling data points

Georeferenced occurrence points of ferns and lycophytes were primarily obtained from the following sources to compile data for analyses. They are as follows: i) occurrences points sampled during field research, detailed below; (ii) voucher records from physical examinations in select herbaria (College of Agriculture Herbarium UP or CAHUP, Los Baños Collection or LBC, & Jose Vera Santos Memorial Herbarium or PUH); and (iii) and historical records accessed via the Global Biodiversity Information Facility database (GBIF, https://doi.org/10.15468/dl.698jtt). Additional occurrence data were harvested from these published works on Philippine ferns and lycophytes: Barcelona and Pelser (2014), Coritico et al. (2019); Amoroso et al. (2020a); Amoroso et al. (2020b); Amoroso et al. (2021); Chao et al. (2022); Chen et al. (2022), and Coritico et al. (2022).

#### 2.1.2 Botanical surveys

**Figure 1:**
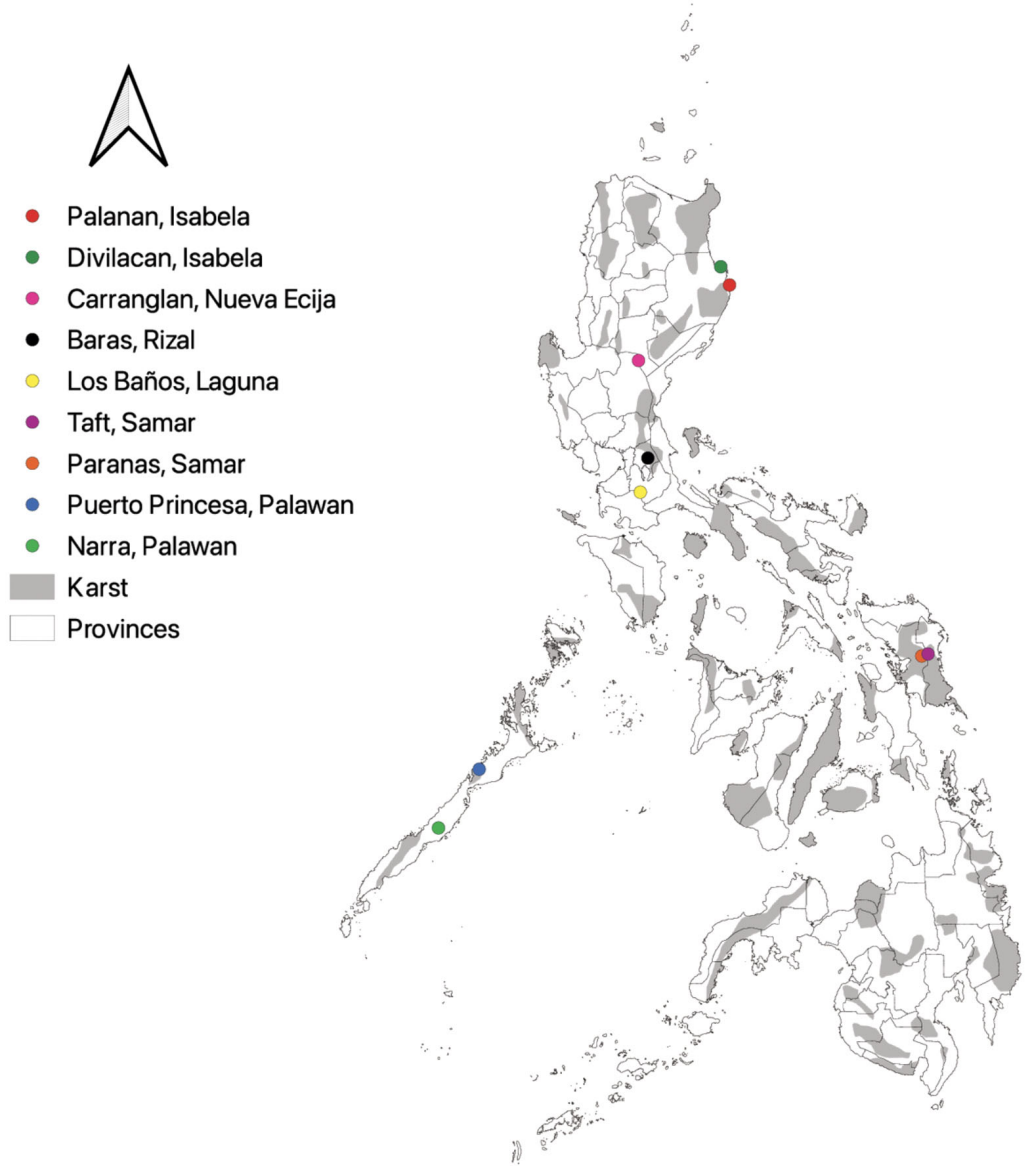
Map showing the survey locations of pteridophytes in Philippine provinces. Gray areas represent karst formations, while lined areas within islands distinguish between and among provinces. Field survey localities include: Palanan, Isabela (red); Divilacan, Isabela (green); Carranglan, Nueva Ecija (pink); Baras, Rizal (black); Los Baños, Laguna (yellow); Taft, Samar (violet); Paranas, Samar (orange); Puerto Princesa, Palawan (blue); and Narra, Palawan (yellow green). Map generated using QGIS 3.32.0 Lima.

As for the field occurrences, the first author conducted a series of field surveys in select provinces of the Philippines from the year 2020–2023. All surveys were carried out with the necessary permissions provided by the administrations overseeing the protection of biodiversity. Both karst areas and non-karst areas were surveyed to account fern and lycophyte species diversity. In total, fieldwork was conducted across nine municipalities from seven provinces of the Philippines, which were 1) Carranglan, Nueva Ecija; 2) Dinapigue, Northern Sierra Madre; 3) Divilacan, Northern Sierra Madre; 4) Masungi Georeserve, Rizal; 5) Mt. Makiling Forest Reserve, Laguna; 6) Puerto Princesa Subterranean River National Park, Palawan; 7) Narra, Palawan; 8) Paranas, Samar Island Natural Park, Samar Island; and 9) Taft, Eastern Samar, Samar Island (**Figure 1**). Species occurrences and geographic locations were recorded using a Global Positioning System (GPS) device, and voucher specimens were deposited at CAHUP and LBC.

#### 2.1.3 Downloading and cleaning occurrence records

Occurrence records on historical Philippine records of ferns and lycophytes were downloaded from the Global Biodiversity Information Facility (GBIF.org, 2023a,b,c,d,e,f,g,h,I,j,k,l,m,n,o,p,q,r,s,t,u,v,w,x,y,z, aa,bb,cc,dd,ee,ff,gg,hh,ii,jj,kk,ll,mm,nn,oo,pp,qq,rr,ss,tt). These data were compiled together with the records obtained from field surveys, herbarium vouchers, and literature records. To ensure the quality of the final dataset, a multi-step occurrence cleaning process was conducted. Those with mismatched described location and GPS coordinates were reassigned based on the description given in the voucher labels. Data points with erratic and incomplete information (e.g., mentioned only country or region names) were excluded. Data points outside the country border were also manually removed.

The species names of the assembled occurrence records were further curated and updated. Family and genus names were standardized in accordance with PPG I (2016), while synonyms were replaced with accepted names based on the nomenclature ascribed in Co’s Digital Flora (Pelser et al., 2011) and World Plants (Hassler, 2004–2023) to reduce transcription errors. In addition, duplicated and misplaced accessions were manually excluded. Finally, spatial autocorrelation between occurrence data was reduced by spatial rarefaction using R package “spThin” v.0.1.0 (Aiello-Lammens et al., 2015) and retained one occurrence point per species within each 1 km^2^ pixel for analyses.

To examine and analyze spatial indices for the two forest formations, the data set was further divided into occurrence records in Karst Landscapes (KL) only and Non-Karst Landscapes (NKL). This was achieved by overlaying the gathered occurrence points with the map of karst formations in the Philippines, then appropriately tagging each occurrence as “KL” or “NKL” based on their location. The Karst map was obtained from the World Karst Aquifer Map available at WHYMAP (World-wide Hydrologeological Mapping and Assessment Programme, 2023), (https://www.whymap.org, downloaded on 9 May 2023).

#### 2.1.4 Assessment of diversity hotspots and priority hotspots

To explore the distribution of ferns and lycophytes in the Philippines, the software Biodiverse version 4.0 (Laffan et al., 2010) was employed to carry out spatial analysis on a grid scale of 0.083333°C (5 arcmin). Three biodiversity indices, namely, (1) species richness (SR), (2) weighted endemism (WE), and (3) corrected weighted endemism (CWE), were calculated for each KL and NKL grid cell. The three indices were defined as follows: (1) species richness represents the sum of the number of distinct/unique species occurring in a grid cell (Chen et al., 2022); (2) weighted endemism, or range-size rarity, assigns high weights to species with small ranges and smaller weights to widespread species (Xue et al., 2022)—with a high weighted endemism value suggesting that there are more range-restricted species in a particular region (Linder, 2001; Huang et al., 2016; Baldwin et al., 2017; Yu et al., 2017); and (3) the corrected weighted endemism, on the other hand, separates the existing trend between weighted endemism and species richness by dividing it by the species richness at each location (Crisp et al., 2001).

To detect the congruence among the distribution patterns of the three indices, we calculated all pairwise Pearson’s correlation coefficients. Pearson’s correlation coefficients (r) were separated into five classes according to Schober et al. (2018): negligible correlation (0 < |r| < 0.1), weak correlation (0.10 ≤ |r |<0.40), moderate correlation (0.40 ≤ |r| < 0.70), strong correlation (0.70 ≤ |r| < 0.90), and very strong correlation (0.90 ≤ |r| < 1). Data cleaning, wrangling, analyses, and visualization were performed in R (v. 2023.06.0 + 421; R Core Development Team, 2023).

#### 2.1.5 Analysis of conservation gaps

To estimate conservation gaps, the protected areas were overlapped with the generated distribution maps based on the three spatial diversity indices. Protected area boundary shapefiles of the Philippines were downloaded from the World Database on (Protected Planet (2014-2023), available at https://www.protectedplanet.net/en/thematic-areas/wdpa?tab=WDPA, downloaded on 1 June 2023). Grid cells within the top 10%, 20%, 30%, and 50% for each spatial diversity metrics were identified as hotspots (Yu et al., 2017). For the gap analysis, the three metrics were overlapped with Philippine protected areas. By overlapping the hotspots resulting from the different metrics, species hotspots and areas that are outside protected area boundaries were visually identified. Maps were generated using QGIS 3.32.0 (QGIS.org, 2023) with WGS-84 EPSG:4326 projection.

#### 2.1.6 Understanding the drivers of distribution between KL and NKL

To understand the drivers of distribution among pteridophyte species from KL and NKL, Principal Component Analysis (PCA) (“PCA” function in the R package FactoMineR) was conducted. Due to the imbalance between KL and NKL points, 2,669 species occurrence points were first randomly selected from the NKL species to match the number of available KL species occurrence points. Analyses were then conducted using three set of parameters: i) all abiotic variables, ii) bioclimatic variables only, and iii) physical/chemical variables only. Bioclimatic variables (BIO1–BIO19) data were obtained from WorldClim 2.1 database at 30 arcs resolution (Fick & Hijmans, 2021). Soil physical/chemical variables were obtained from World Soil Information (ISRIC) at 250 m resolution then resample to 30 arcs. Abiotic variable values were extracted for each species occurrence point. Values were then normalized prior to the PCA run.

Predictor variables that had correlations with response variables between 0.1 and −0.1 due to weak explanatory power were removed (Fløjgaard et al., 2011). On account of collinearity, when two or more predictor variables were strongly correlated (r > ± 0.70), we only retained variables that had the stronger correlation with the response variable and makes biological sense with the taxa (Dormann et al., 2007). Data was analyzed using the software R, version 2023.06.0 + 421 (R Development Core Team, 2023).

## 3 Results

### 3.1 Fern and lycophyte species diversity in the study region

A total of 62,887 occurrence points were compiled by incorporating 302 physical voucher examinations (PUH, LBC, and CAHUP), 683 field derived coordinate points, and 61,902 GBIF-derived occurrence data. Of the 1,430 species, a total of 406 were excluded from the analysis due to erratic and/or controversial information such as unplaced names, no formal species records, or incorrect distribution occurrence records. A total of 19,259 points remained after spatial thinning and were utilized for analysis (**Supplementary Table 1**). In total, there are 1,024 fern and lycophyte species, 992 species of which were in non-karst areas. Out of the 679 species recovered to occur in karst areas, 5% were found only in karst and 95% in both karst and non-karst areas (Fick and Hijmans, 2017).

### 3.2 Spatial patterns of fern and lycophyte species diversity and hotspots in karst landscapes in the Philippines

**Figure 2:**
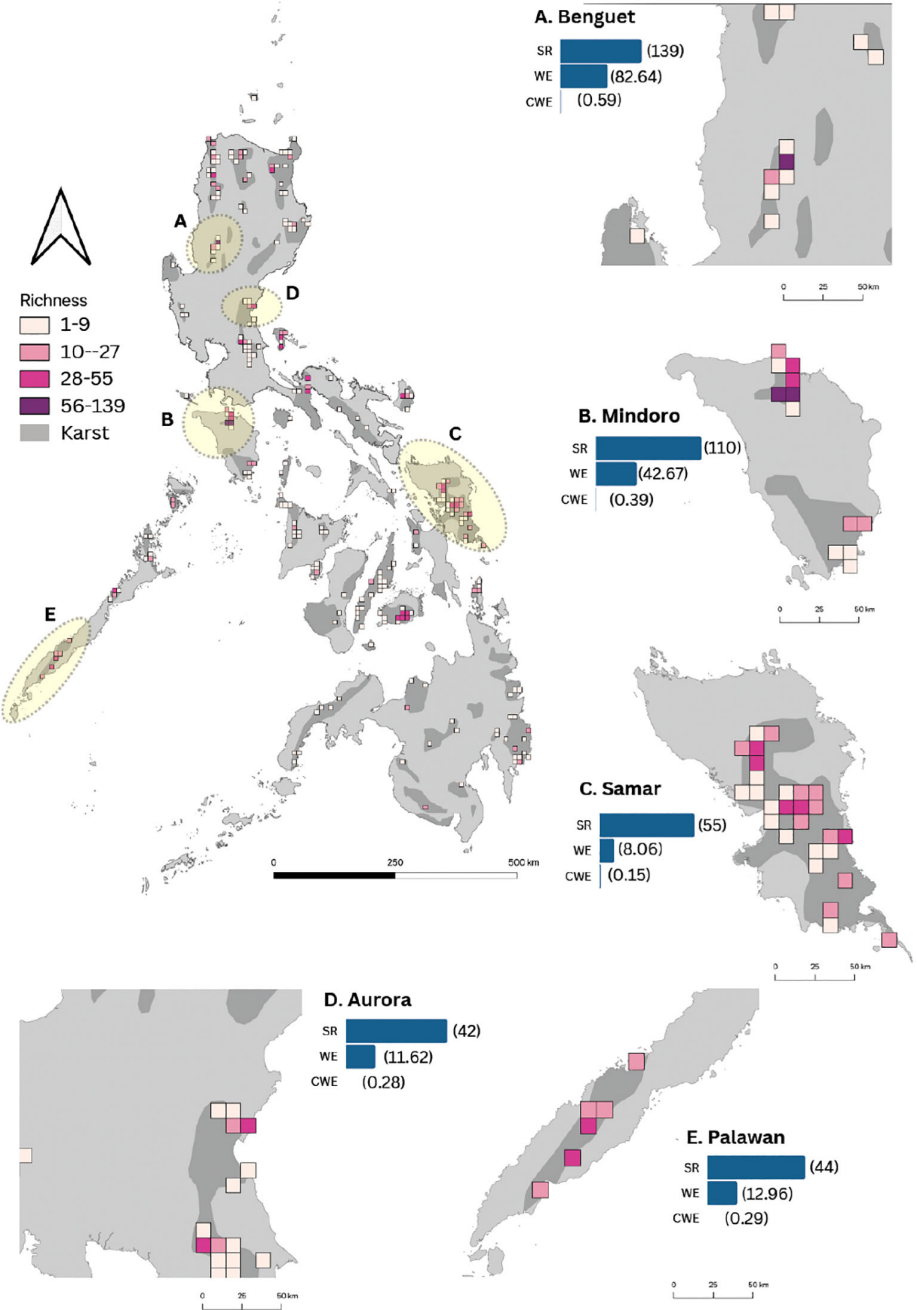
Distribution of fern and lycophyte species richness and endemism in karst habitats across the Philippines. Karst formations are shaded in gray. Provinces with high species richness and endemism are highlighted in yellow and zoomed in. The color code represents the four classes of species richness: Class I (1–9 species) in light pink, Class II (10–27 spp.) in pink, Class III (28–55 spp.) in dark pink, and Class IV (56–139 spp.) in purple. The identified hotspot provinces **(A–E)** are accompanied by bars indicating species richness (SR), weighted endemism (WE), and corrected weighted endemism (CWE). Numbers in parentheses correspond to the index values for SR, WE, and CWE.

The reconstructed fern and lycophyte species spatial patterns in karst areas reveal 10 hotspot areas. The distributional range of species occurring in karst formations of the Philippines comprised of 2,669 occurrence records in 248 grid cells. SR per grid cell ranged from 1 to 139 species (**Figure 2**). Most of the karst landscape identified to have fern and lycophyte species rich flora were found at the foot or near mountain ecosystems. Species-rich provinces comprised i) Kabayan, Benguet; ii) Baco, Mindoro Oriental; iii) San Teodoro, Mindoro Oriental; iv) Paranas, Samar; v) Hinabangan, Samar; vi) Dingalan, Aurora; vii) Samarenana, Palawan; viii) Caramoan, Catanduanes; ix) Valencia, Bohol; and x) Guinayangan, Quezon. A consistent pattern was observed by exploring the spatial distribution for WE. The spatial distribution of SR was highly correlated with WE (R = 0.95). In turn, SR and WE had a weak correlation with CWE (R = 0.19; R = 0.25). Notably, records were rare or totally missing for six provinces with karst formations in the Philippines. These areas were identified to be Davao del Norte, Masbate, Quirino, Saranggani, Southern Leyte, and Surigao del Sur. Five from these 10 landscapes have existing natural parks and other protected landscapes.

### 3.3 Spatial patterns of fern and lycophyte species diversity and hotspots in non-karst landscapes in the Philippines

**Figure 3:**
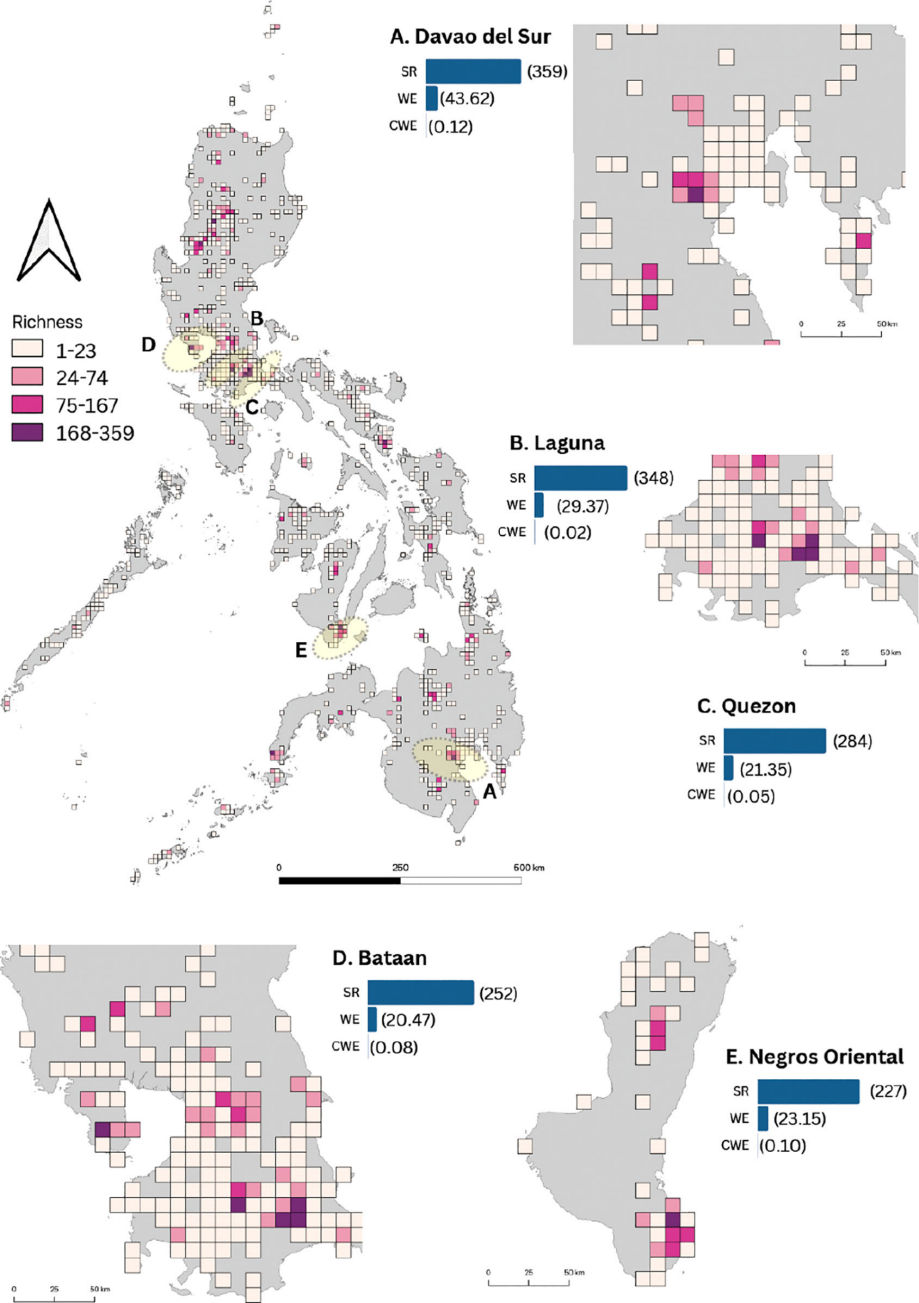
Distribution of fern and lycophyte species richness and endemism in non-karst areas across the Philippines. Provinces with high species richness and endemism are highlighted in yellow and zoomed in. The color code represents four classes of species richness: Class I (1–23 spp.) in light pink, Class II (24–74 spp.) in pink, Class III (75–167 spp.) in dark pink, and Class IV (168–359 spp.) in purple. The maps of these five provinces **(A–E)** are accompanied by bars indicating species richness (SR), weighted endemism (WE), and corrected weighted endemism (CWE) for the respective province. Numbers in parentheses represent values for SR, WE, and CWE.

The final spatial dataset for fern and lycophyte occurrences in non-karst areas contained 16,590 occurrence points in 854 grid cells. SR per grid cell ranged from 1 to 359 species (**Figure 3**). Species-rich provinces consisted of i) Santa Cruz, Davao del Sur; ii) Los Baños, Laguna; iii) Candelaria, Quezon; iv) Bagac, Bataan; v) San Jose, Negros Oriental; vi) Lucban, Quezon; vii) La Trinidad, Benguet; viii) Tayabas, Quezon; ix) Bauko, Mountain Province; and x) Casiguran, Sorsogon. Existing natural parks and other protected landscapes were in all these regions.

These provinces were recognized for the presence of ecologically and culturally significant mountain ecosystems including some dormant volcanoes. Mountain ecosystems representing putative cradles or sanctuaries of diversity included protected landscapes such as i) Mt. Apo National Park (MANP) in Davao del Sur, ii) Mt. Makiling Forest Reserve (MMFR) in Laguna, iii) Mt. Banahaw-San Cristobal Protected Landscape (MBSCPL) in Quezon, iv) Mt. Mariveles in Bagac, Bataan, v) Balinsasayao Twin Lakes Natural Park, Negros Oriental, vi) Mt. Santo Tomas, Benguet, vii) Mt. Polis, Mountain Province, and viii) Bulusan Volcano National Park, Sorsogon. Like the results of KL, the spatial distributions of NKL for SR was highly correlated with WE (R = 0.80). However, SR and WE had a weak correlation with CWE (R = 0.07; R = 0.29).

### 3.4 Hotspots and conservation gaps of Philippine fern and lycophyte species occurring in karst and non-karst landscapes in the Philippines

Based on the diversity metrics SR and WE indices, the top 10% hotspots were concentrated in Batan, Benguet located in Northern Luzon. Extending the selected hotspots to the top 20% revealed by SR and WE expanded the list towards Mangan II and Caagutayan of Mindoro Oriental. Further extending to the top 30% included (1) Paranas and Bagacay, Samar; (2) Valencia, Bohol; (3) Samerana, Palawan; (4) Dikapanikian, Aurora; (5) Caramoan, Catanduanes; (6) Guinayangan, Quezon; (7) Bilar, Bohol; (8) Gandara, Samar; and (9) Tagkawayan, Quezon. Finally, when expanded further to 50%, grid cells with high values for SR and WE now included also (1) Carmen, Bohol; (2) San Teodoro, Mindoro Oriental; (3) Rizal, Palawan; (4) Verde Island Passage; (5) Cabayugan, Puerto Princesa, Palawan; (6) Sierra Bullones, Bohol; and (7) Nueva Era, Ilocos Norte. Most of the hotspot areas for SR and WE were concentrated in the Luzon and Visayas islands.

**Figure 4:**
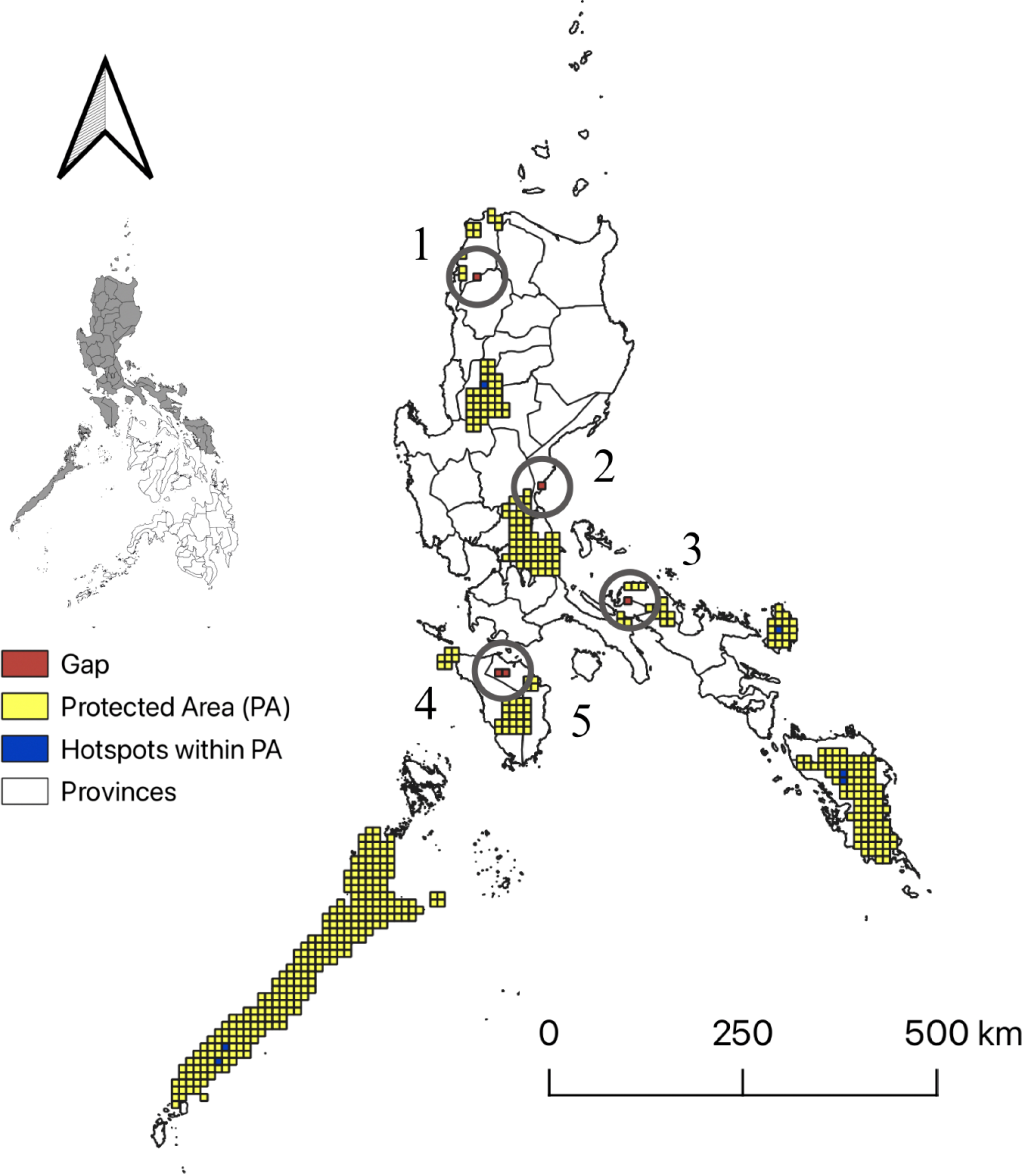
Hotspot areas and gap areas of pteridophyte species occurring in forests formed over karst formations. Hotspots were defined as the overlap of the richest 50% of grid cells for species richness (SR), weighted endemism (WE), and corrected weighted endemism (CWE). Hotspots inside protected areas are shown in blue, while hotspots outside protected areas are shown in red. Identified gap areas (red) include 1) Nueva Era, Ilocos Norte; 2) Dikapanikian, Aurora; 3) Bagong Silang, Tagkawayan, Quezon; 4) Mangangan, Baco, Mindoro Oriental; and 5) San Teodoro, Caagutayan, Mindoro Oriental. Map generated using QGIS 3.32.0.

Most of the differences in overlap among these diversity indices were found at 50%. Once 50% was considered as the extent of congruence, results revealed more unique hotspots and was thus chosen as the ideal threshold for defining pteridophyte hotspots. A total of 11 areas were identified as hotspot areas. These congruent hotspots cover approximately 93.5 km^2^. Six of the 11 congruent hotspot areas are found within protected areas: 1) Batan, Benguet; 2) Samarenana, Palawan; 3) Caramoan, Catanduanes; 4) Gandara, Samar; 5) Rizal, Palawan; and 6) Matalud, Samar. However, the five other areas that were identified to be congruent hotspots, now considered as gap areas, that are not within protected areas include the following: 1) Nueva Era, Ilocos Norte; 2) Dikapanikian, Aurora; 3) Bagong Silang, Tagkawayan, Quezon; 4) Mangangan, Baco, Mindoro Oriental; and 5) San Teodoro, Caagutayan, Mindoro Oriental (**Figure 4**).

### 3.5 Drivers of fern and lycophyte assemblage in karst and non-karst landscapes

**Figure 5:**
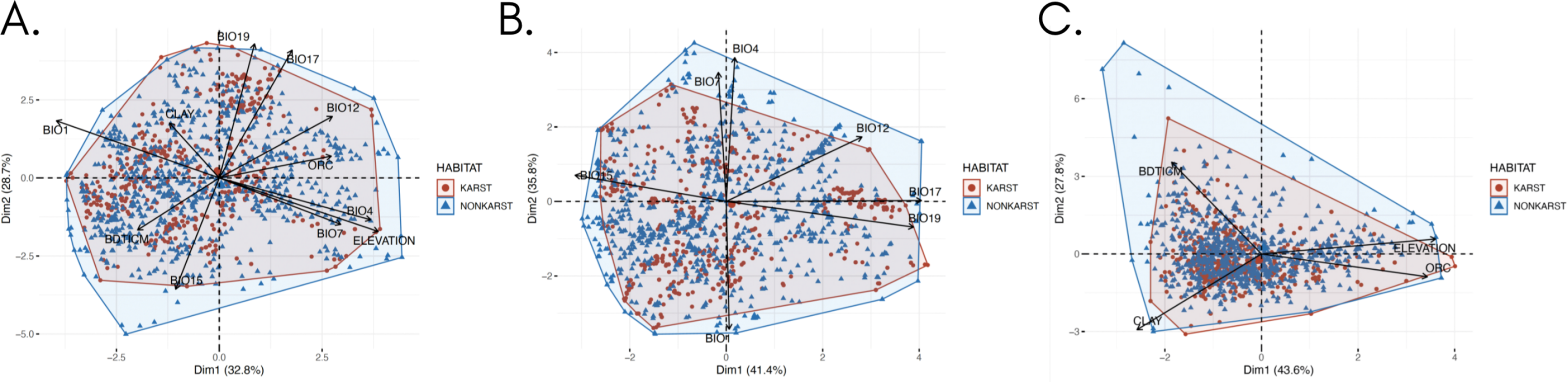
Principal components analysis (PCA) plots illustrating the influence of **(A)** all variables, **(B)** bioclimatic variables, and **(C)** soil variables on the distribution of ferns and lycophytes within Philippine ecospace. The scatterplot represents a PCA focused on dimensions 1 and 2, with data points color-coded by habitat: blue for karst and red for non-karst. Vectors represent the variables: BIO1, BIO4, BIO7, BIO12, BIO15, BIO17, BIO19, absolute depth to bedrock (BDTICM), elevation, organic carbon content (ORC), and clay content.

Principal component analyses did not reveal influential factors driving species distribution between karst and non-karst areas, as there was no discernible clustering between karst and non-karst species occurrence points (**Figure 5** and **Supplementary File 3**). The PCA, which considered bioclimatic variables alone, explained the highest proportion of variance (77.2%) (**Supplementary File 10B** and **Supplementary File 6**), followed by the PCA that used soil and elevation variables (71.4%) (**Supplementary File 10C** and **Supplementary File 8**). Despite the identification of these explanatory variables, these were insufficient to distinctly separate ferns and lycophytes into habitat groups of karst and non-karst landscapes, with ferns and lycophytes recorded from karst landscapes nested within non-karst landscapes.

## 4 Discussion

Limited studies have been conducted to explore the composition of ferns and lycophytes in karst landscapes of the Philippines. The challenging terrain and limited accessibility to these landscapes have significantly hindered botanical exploration. This has resulted in a sampling bias that is evident in the disproportionately higher number of species occurrence records documented from non-karst landscapes. To avoid underestimating ferns and lycophytes species associated with karst environments, it is crucial to conduct more fieldwork. This involves direct documentation of plant specimens in relation to their substrate complemented with the integration of other data sources.

### 4.1 Lack of data in karst landscapes of the Philippines

To address data gaps due to the lack of targeted surveys in karst areas, this study used an approach that considers available voucher specimens available and historical data from GBIF. However, unlike that of field-derived data and publications with substrate information, only a limited number of vouchers and historical data contains information regarding the substrate. Thus, the association between plant and substrate are not directly accessible by the collector information. To overcome this limitation, we employed a mapping approach that overlays plant and karst distribution maps to infer associations.

Conducting thorough assessments in karst landscapes presents challenges, as evidenced by the bias towards collecting data from non-karst landscapes. The higher number of species occurrences recorded in NKL may be a consequence of the rugged terrain, topographic isolation, and limited accessibility of karst areas in the Philippines. Additionally, the available edaphic, topographic, and bioclimatic data online is coarse and could be insufficient in capturing changes in microenvironments of karst landscapes. We suspect that the low-resolution data available and used for the PCA could be the reason why no distinct clusters between the two environmental types were apparent. Future related work should consider using field-derived environmental data for ecological analysis. Spatial heterogeneity in soil nutrients and moisture along karst ecosystems, influenced by the rate of karst formation and carbonate rock substrate, highlights the need to gather detailed abiotic data directly from fieldwork (Geekiyanage et al., 2019). Field data play a crucial role not only in providing precise location information but also in helping to understand ecological processes underlying in karst formations that distinguishes them from their non-karst counterparts and in confirming the relationship between fern and lycophyte species and their substrate.

### 4.2 Not all “landscapes” are protected in equal measure: a need for expanding conservation strategies to karst landscapes

The foundation of effective conservation decision-making relies on the accuracy and precision of the data used to design present and future management strategies (Hughes et al., 2021a, Hughes et al., 2021b). It is essential to formulate data-driven conservation strategies guided by robust scientific principles. Protected areas, both existing and prospective, represent a key strategic approach for biodiversity preservation (Sodhi et al., 2004; Matten et al., 2023). Our analysis identified 11 karst landscapes as critical hotspots for pteridophytes, with the majority of these areas falling within protected area boundaries. While these areas will remain protected for the foreseeable future, some karst areas, such as limestone forests, remain inadequately safeguarded. Through gap analysis, previously overlooked areas requiring conservation intervention were identified. Notably, five of these 11 hotspots areas lack any legal protection, underscoring the urgency to expand the adjacent protected areas to cover these crucial karst regions, thus safeguarding the pteridophyte species they harbor.

The conservation of these areas not only ensures the preservation of karst landscapes and their associated pteridophytes but also safeguards the numerous ecosystem services these unique forest formation provides. The success of the Kunming Montreal Biodiversity Framework (CBD, 2022) depends largely on our knowledge and understanding of plant diversity and distribution; in its absence, there is an overwhelming risk that some of the global commitments—such as pledge to protect 30% of the world’s terrestrial surface by 2030, potentially escalating the risk of plants species loss (Antonelli, 2023). Existing protected areas and future protected areas remain to be one of the most strategic and effective means of preserving biodiversity (Sodhi et al., 2004; Matten et al., 2023).

To enhance conservation efforts, it is crucial to promote the growth and training of experts capable of species identification and proficient in collating high-resolution biological, ecological, and spatial-temporal species data. Establishing standardized long-term monitoring strategies for ferns and lycophyte species is recommended to ensure data reliability and track secure species populations in the wild. Continuous inventories, spatial occurrences, and comparative studies of fern communities are pivotal in bridging biodiversity information gaps and preventing extinctions (Pimm, 2020). A robust database of species occurrences is instrumental in predicting community patterns and determining conservation priorities for ferns and lycophytes in both karst and non-karst landscapes.

## 5 Conclusion

This study utilized available ferns and lycophytes occurrence data from multiple sources to visualize spatial diversity of ferns and lycophytes between karst and non-karst landscapes. We demonstrated that fern and lycophyte hotspots are concentrated in mountain ecosystems within established protected areas. However, we also found that historical records available are unevenly distributed across the Philippines and biased towards non-karst landscapes, resulting in survey gaps concentrated on the karst formation of five areas. This study underlines the need to provide special protection for tropical karst forest ecosystems because of their unique species assemblages, many of which exhibit localized distributions and are highly susceptible to habitat loss from mining and other anthropogenic activities. Furthermore, this study proposes to promote the expansion of protected areas to accommodate identified fern and lycophyte gap areas within karst landscapes. This proactive approach aligns with the imperative to conserve and sustainably manage these ecologically valuable habitats for the benefit of both biodiversity and local communities.

## Data Availability

The original contributions presented in the study are included in the article/**Supplementary Material**. Further inquiries can be directed to the corresponding author.
