# Supplementary figures and images for: Spatial diversity and distribution of fern and lycophyte species in karst and non-karst landscapes towards conservation needs
Source: Front Plant Sci. 2025 Mar 3;16:1495796. doi: 10.3389/fpls.2025.1495796 (PMC11912880; doi:10.3389/fpls.2025.1495796)

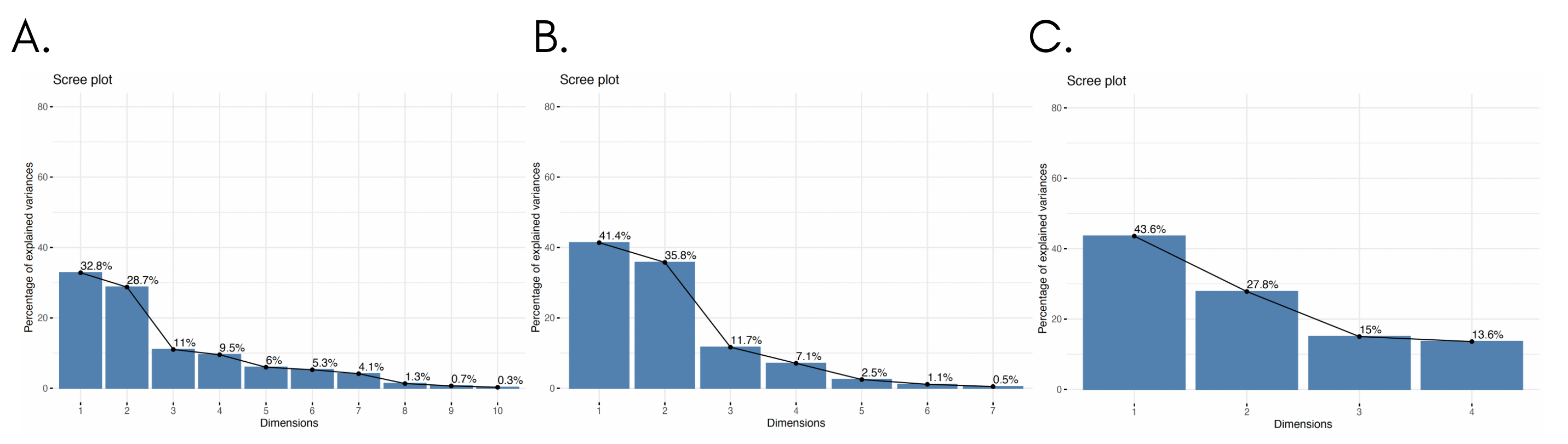

Supplement: Supplementary file 10 [file Supplementaryfile10.png]
